# Effect of Shear and Pure Bending Spans on the Behaviour of Steel Beams with Corrugated Webs

**DOI:** 10.3390/ma15134675

**Published:** 2022-07-03

**Authors:** Ibrahim A. Sharaky, Yasir M. Alharthi, Ahmed S. Elamary

**Affiliations:** Civil Engineering Department, College of Engineering, Taif University, P.O. Box 11099, Taif 21944, Saudi Arabia; i.sharaky@tu.edu.sa (I.A.S.); y.harthi@tu.edu.sa (Y.M.A.)

**Keywords:** corrugated web, steel beams, finite element model, shear span, bending span

## Abstract

The shear span-to-effective depth ratio is known to modulate the shear behaviour of steel beams with corrugated webs (SBCWs). However, present design standards for SBCWs do not adequately address this issue. The impact of shear span-to-effective depth ratio and pure bending spans on the failure mechanism of SBCWs was investigated in this study. Under four-point bending, three beams with shear-span-to-effective-depth-ratios ranging from 1.65 to 2.5 were examined to investigate the relationship between shear and bending spans and failure mechanisms. ANSYS software was used to create finite element models for the tested SBCWs using the finite element technique. In addition, the experimental findings are compared to two codes, specifically DASt-Rishtlinie015 and EN 1993-1-5. Moreover, an analytical section comprised of the creation of a three-dimensional (3D) finite element model (FEM) was implemented. Finally, a parametric study using the verified FE model was conducted to assess the impact of shear and pure bending spans on the overall behaviour of SBCWs. As a result, the shear span and horizontal fold length of CWSBs are key components for determining the strength and failure modes of beams. Furthermore, the load capabilities and stiffness of CWSBs were more greatly affected by increasing the shear span than by increasing the pure bending one.

## 1. Introduction

Steel beams with corrugated webs (SBCWs) have thin webs and no transversal stiffeners, giving them an advantage over flat web beams (FWB) in terms of cost and weight. However, because of its geometric properties, CWB girders have a few weaknesses, which may be divided into two categories. First, increasing the flange’s outstand length improves its slenderness, resulting in the flange developing local buckling strength earlier than an FWB [1]. Second, due to web eccentricity, an extra in-plane transverse moment develops in the flange [2,3], which greatly reduces the CWBs’ flexural capacity. SBCWs have been widely examined in terms of shear and bending strength. Elgaaly et al. [4] conducted worthwhile experimental and analytical studies, mostly applying loaded to shear. To estimate the SS of SBCWs, Abbas et al. [5] and Sause et al. [6] performed large-scale SBCW studies. Previous research [1,2,3,4,5,6,7,8,9,10] showed that when steel beams are subjected to in-plane shear and bending, the FW has a lower shear capacity than the SBCWs. Furthermore, when bending and shear forces are applied to CWB steel girders, the two forms of displacement that occur concurrently (in-plane and out-of-plane) in the CWB are assigned to two phases of failure—the first is driven by flange yield stress, while the second is controlled by web shear stress. Furthermore, these experiments showed that the geometry of the web profile has a direct relationship with the variable of flange transverse bending moments in a CWB. In the previous three decades, the bending and shear behaviour of SBCWs has been intensively researched [11,12,13,14,15,16,17,18,19,20], particularly in terms of shear behaviour. To forecast local, global, and interaction shear buckling, formulas have been developed. Abbas et al. [3] found that in-plane flexural stresses, along with flange transversal displacing stresses, are applied as normal stress on the flange cross section as confirmed in [20,21,22].

Earlier literature on SBCW web and flange buckling [3,4,5,6,7,8,9,10] looked at the interaction between local and global shear stress, web slenderness, initial geometric faults, geometrical frequency, panels breadth, web (thickness and height) and steel grade. In coarsely and dense corrugations, local and global web buckling was observed, respectively. They also demonstrated that, until the web fractures, shear force is equally distributed over its height. CWB also offers better shear stability and fatigue resistance than a typical flat-web beam, according to various studies [11,12,13,14,15,16,17]. Some of these investigations [18,19] concentrated on determining the bending resistance of composite SBCWs. These tests revealed that the flexural and shear behaviours of composite SBCWs had no correlation. Kövesdi et al. [21] evaluated six large-scale test samples with trapezoidal corrugated webs in an experiment. Two samples were tested in a four-point layout, but most were broken in a three-point pattern. Three-point bending, four-point bending, and evenly distributed load bending were all quantitatively evaluated in this study. They drew the conclusion that, under combined bending and shear loads, the normal stress distribution in the flange is dependent on the analysed specimen geometry, load location, and support position. As a result, an improved design equation for determining the maximum transverse bending moment was developed. Other research [22,23,24] looked at the shear and bending resistances of steel girders using FWBs. Dabon and Elamary [25] used both experimental and analytical methods to examine the flexural capabilities of steel beams with corrugated and flat webs. They found that the FWB had a larger capacity than the CWB under a pure bending moment.

The shear-span ratio of CWSBs has a significant impact on buckling development and failure mechanism. Despite extensive studies on the shear and bending buckling resistances of flat and corrugated web girders, the impact of changing shear and bending spans, and web horizontal fold length on beam capacity has been subject to a few investigations. Based on maximum load capacity and deflection, the basic concept is to investigate the influence of shear span with variable web profile geometry (i.e., horizontal fold length) throughout the length of the beam. The current study investigates the failure mechanism of numerous steel beams with varying shear spans and web-profile geometries along the beam length using both experimental and analytical methodologies. In four-point bending, three SBCWs with dimensions of 200 × 400/500 mm × 2450 mm, varying shear and pure bending spans, and various highs were tested. Beams with an effective shear span depth ratio between 1.65 and 2.5 were tested. Three specimens with varying shear spans and web forms were fabricated as follows: one had a shear span that was 1.5 times the bending span, another had a shear span that was equal to the bending span, and the third had both shear and bending spans that were equal and a deeper web depth. The beams were subjected to displacement-controlled loads, and the mid-span deflections of the beams were recorded using a data acquisition system until failure. The strength variations as a function of shear span to depth ratio and pure bending span were obtained and compared. The buckling of the flange and web was observed and quantified during testing at all load levels until failure. The authors’ experimental results were compared with predictions from available design guidelines. Two derived formulae given by various standards were used to calculate the expected moment capacity of the specimens to point out the most appropriate one that might potentially be used. A three-dimensional finite element model was built to simulate the specimens analytically. For validating the 3D FE model, the tested outcomes along with the experimental data were compared with the model results. Parametric statistics were also carried out to see how the lengths of shear and bending spans affected CWB behaviour.

## 2. Experimental Program

### 2.1. Fabrication and Details of Specimens

Under four-point bending, three full-scale steel beams were tested. In the three specimens, different shear span, pure bending span, and web height combinations were produced. The first specimen was made with shear spans greater than the pure bending span, whereas the second and third specimens were made with shear spans equal to the pure bending span. The three specimens’ first and third panels were designed to be in the bending moment and shear force areas, respectively, while the middle panel was designed to be in the flexure zone. The first had a shear span of 900 mm and a pure bending span of 600 mm. The second had a shear span of 800 mm and a pure bending span of 800 mm. The web height of both the first and second specimens was 400 mm. The third example was constructed with the same shear span and pure bending parameters as the second, but with a web height of 500 mm. S90B60h40, S80B80h40, and S80B80h50 are the specimens’ designations.

**Figure 1:**
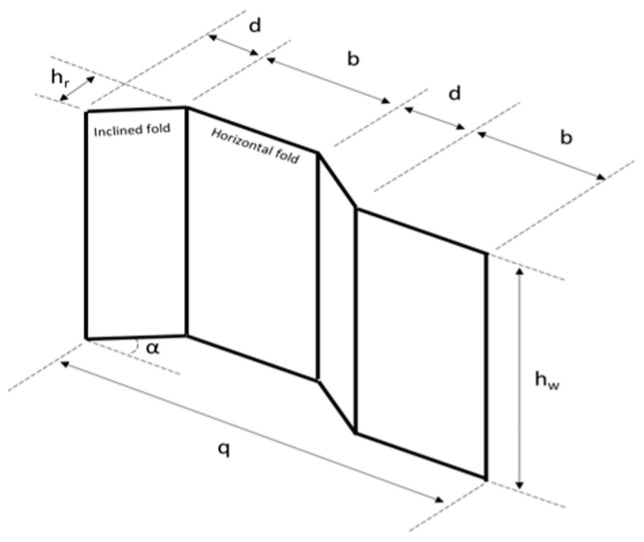
Corrugated web profile configuration.

The tested specimens had an effective span of 2400 mm and a length of 2450 mm. The web thickness (*t_w_*), flange breadth (*b_f_)*, and flange thickness (*t_f_*) were all 3, 200, 8 mm. The web height (*h_w_*) was also changeable. The determined height-to-thickness ratio (*h_w_*/*t_w_*) of the corrugated web for the first and second specimens was 128 and it was 160 for the third specimen. The compactness of the flanges in the tested specimens was assessed with regard to the maximum outstanding length, i.e., (*h_r_* + *b_f_*)/2*t_f_* = 18.75, since the flange compactness measured for a corrugated-web beam (i.e., the outstanding length) was changeable throughout the inclined panel length. Based on the slenderness ratios of the web and flange, the section might be classed as a Class 4 thin section according to the EN 1993-1-5 [26] section categories. The inclined fold’s corrugation depth (h) and horizontal projected length (d) were both 100 mm. The angle of corrugation was 45°. The corrugation profiles of the specimens are shown in Figure 1.

Four steel-plate stiffeners (384/484 mm 200 mm 8.0 mm) were employed for each specimen: two as bearing stiffeners over the supports and two as concentrated load stiffeners. The DIN-EN 1011-2 standard [27] was followed for welding the built-up section, bearing, and load stiffeners together. Welding safety standards were followed to minimize beam distortion caused by the high temperature of the welding process, which was especially important for the thin sections. The measured parameters were denoted by a code, where “S,” “B,” and “h” indicated for “shear span,” “pure bending span,” and “specimen high,” respectively. Each specimen’s details are listed in Table 1.

**Figure 2:**
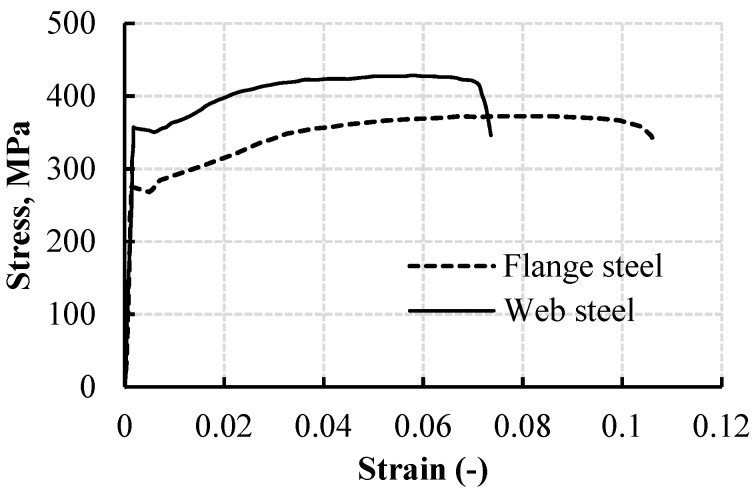
Flange and web strength and elongation strain.

To evaluate the web and flange steel properties, three typical specimens (300 mm length) were taken and manufactured from each CWSB specimen. The coupons were machined to 0.01 mm precision. Following that, the coupons were evaluated in line with the EN 10002-1 standard [28]. The obtained tensile properties of the web and flange materials are listed in Table 2. The specimens’ stress–strain responses are displayed in Figure 2.

### 2.2. Testing Procedure and Instrumentations

**Figure 3:**
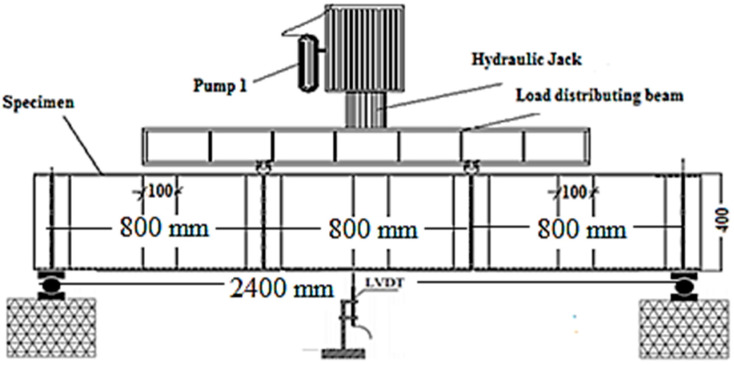
Test set-up.

A four-point bending load was applied to all of the CWSBs that were evaluated, with a shear span of 800 mm or 900 mm (Figure 3). A loading frame with a 2000 kN hydraulic jack was used to apply the load automatically at a displacement rate of 0.6 mm/min. One linear variable differential transformer was used to measure the mid-span vertical deflection. A data capture software directly connected to a computer was used to acquire the measured data every second.

### 2.3. Test Results and Discussion

Based on the EC3-EN1993-1-1- Table 5.2, the specimen cross sections are classed as class 4, indicating that buckling will be performed locally where the yield stress was not reached. Table 3 shows the buckling and maximum load capacities (*P_k_* and *Pu*), as well as buckling and ultimate loads and deflections, with the corresponding failure mechanisms of the tested beams.

**Figure 4:**
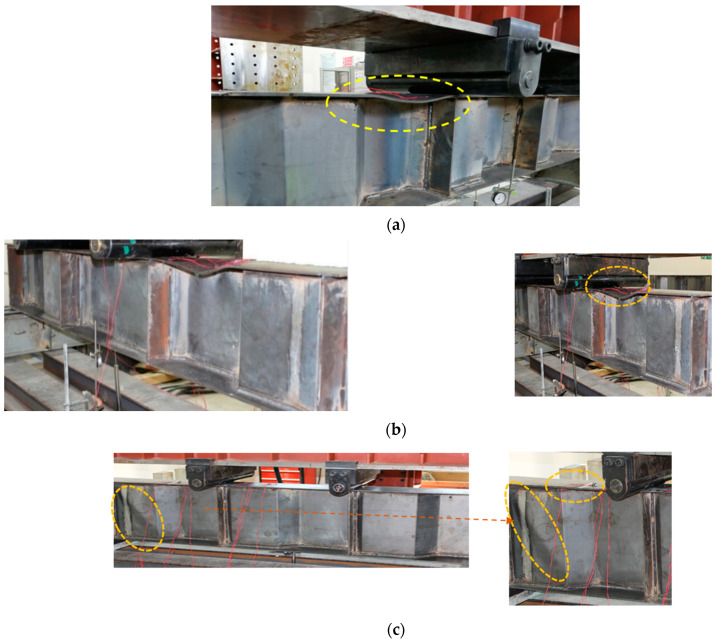
Failure mode of Specimens (**a**) S90B40h40 (flange buckling at M-V zone); (**b**) S80B80h40 (flange buckling at M-V zone); (**c**) S80B80h50 (Web buckling at M-V zone).

As demonstrated in Figure 4a,b, specimens S90B60h40 and S80B80h40 both failed owing to top flange local buckling (FB). This suggests that FB is the principal buckling mechanism in control of S90B60h40 and S80B80h40’s overall behaviour in the M–V zone. The influence of TBM described in the introduction is highlighted by flange buckling of the beams in the moment shear zone rather than the pure flexural zone. Meanwhile, web shear buckling (WB) is implicated for S80B80h50’s failure (Figure 4c), which is followed by exceptionally low FB in the M–V zone.

#### Load-Deflection Response

**Figure 5:**
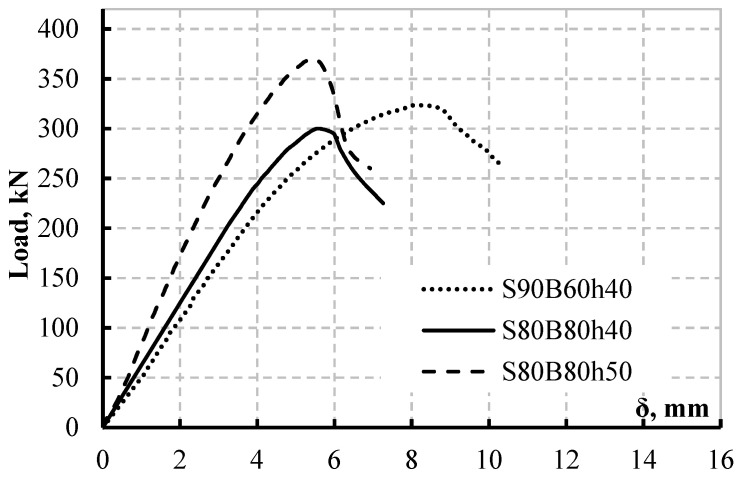
Mid-span vertical deflection—vertical loads of the tested beams.

**Figure 6:**
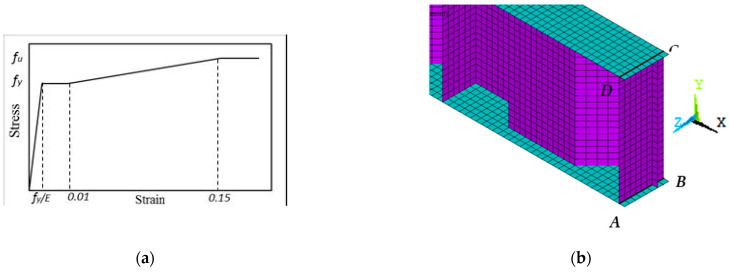
Finite element modelling, (**a**) Material, (**b**) Boundary Conditions.

Based on four-point bending tests and observations, this part discusses the behaviour of the studied specimens with respect to load deflection response and stiffness. Figure 5 shows the load–deflection (P–δ) responses of the CWSB specimens. The highest deflections achieved by the three specimens are also compared in Figure 5. Different load–deflection responses were seen in the three specimens, as was the maximum deflection. At a deflection value (δ) of nearly 8.25 mm, specimen S90B90h40 attained its ultimate load, whereas specimens S80B80h40 and S80B80h50 reached their maximum loads at = 5.79 mm. As can be shown, CWSBs with a web height of 400 mm have almost comparable initial stiffness regardless of the length of shear and bending spans. All of the CWSBs examined had P–δ curves with comparable properties that may be separated into two phases. Before buckling, the beams had a nearly linear P–δ relationship in the first phase (from zero load to buckling). Phase 2 shows that, after yielding, the beams’ stiffness decreases somewhat, and the top flanges or web buckle as the force is greater. As the load decreases somewhat, the deflection of the CWSBs dramatically rises. The loading locations with regard to the shear and bending span have a minimal influence on the initial stiffness of the beams for CWSBs of the same height. The rigidity of the beam increases dramatically by increasing the height of the web from 400 to 500 mm (Figure 6). The web height affects the stiffness of the beams in the same way that it affects the load capacities of the CWSBs, implying that the web height can not only enhance beam stiffness and capacity but also influence the failure mechanism. Theoretically, when the shear span increases, CWSBs with the same material properties should have a lower maximum load value; nevertheless, the findings of specimens S90B60h40 demonstrate a higher capacity and maximum deflection than specimen S80B80h40. This leads to the conclusion that the application of the load over CWBs is impacted by the HFs’ length more than the shear span, according to the loading deflection curves scenario. According to Figure 5, CWSBs with flange buckling control perform better in terms of ductility than those with web buckling control, based on overall behaviour and failure cause.

## 3. Theoretical Analysis

Based on the equations recommended by two codes EN 1993-1-5 [26] and DASt-Rishtlinie015 [29], the flexural capacity of SBCWs is evaluated as described in this section.

### 3.1. EN 1993-1-5 Standard

Equation (1) might be implemented to compute the bending resistance according to EN 1993-1-5 Annex D [26], with appropriate safety for flange buckling.

(1) $$M_{Rd}=min\left[\begin{array}{c} \left(\frac{b_{uf}*t_{uf}*f_{yf,r}}{\gamma_{M0}}*\left(h_{w}+\frac{t_{uf}+t_{lf}}{2}\right)\right);\\ \;\left(\frac{b_{lf}*t_{lf}*f_{yf,r}}{\gamma_{M0}}*\left(h_{w}+\frac{t_{uf}+t_{lf}}{2}\right)\right);\\ \;\left(\frac{b_{uf}*t_{uf}*\chi *f_{yf}}{\gamma_{M0}}*\left(h_{w}+\frac{t_{uf}+t_{lf}}{2}\right)\right) \end{array}\right],$$

where *t_uf_* and *t_lf_* are the top and bottom flange thicknesses, respectively; *h_w_* is the web height; *f_yf_* is the yield stress of the flange; *ɤM*_0_ is the factor of safety and is the buckling reduction factor. According to EN 1993-1-5 Annex D [26], the bending resistance of CWSBs subjected to shear and flexural should be regulated by a reduction factor. From the transverse bending moment the maximum normal stress level can be determined, further to the yield stress of the flange; the reduction factor (*f_T_*) can be evaluated as stated in Equations (2) and (3). The lower bending resistance is calculated as follows:

(2) $$f_{yf,r}=f_{T}*\;f_{yf}$$

(3) fT=1−0.4∗σx,(Mz)fyfγM0=1−0.4∗(6∗Mztf∗bf2)fyfγM0

(4) $$M_{z}=\frac{V_{z}*h_{r}}{4*h_{w}}\left(2*b+d\right)$$

(5) $$\bar{\lambda }=\sqrt{\frac{A*f_{y}}{N_{cr}}}=\frac{L_{cr}}{i}*\frac{1}{\lambda_{1}}$$

(6) $$\lambda_{1}=\pi *\sqrt{\frac{E}{f_{y}}},$$

where $f_{yf,r}$ is the magnitude of the yield stress decreased according to transverse moments in the flanges, $\sigma_{x,\left(M_{z}\right)}$ is the stress due to the transverse moment coming from shear flow in the flanges, and $\gamma_{M0}$ is the stress assessment partial factor. Kövesdi et al. [21] suggested the approximate formula for $M_{z}$ (Equation (4)) as shown in Equation (7). According to Kövesdi et al. [21], the maximum reduction factor value is not more than 7.2 percent.

(7) $$M_{z}=\frac{V_{z}*h_{r}}{2*h_{w}}\left(2*b+d\right),$$

where χ is the reduction factor created by Lindner [2]; it is also included in the German guidelines (DASt-Richtlinie 015, [29]). The EN 1993-1-5 discussion paper [26] likewise notes that these bending moments are essential for equilibrium from a theoretical standpoint, but their practical significance is debatable. $\bar{\lambda }$ is the slenderness ratio-based reduction factor for out-of-plane buckling (6.3 EN1993-1-1), A is the cross-section area, $N_{cr}$ is the flexible essential strength for the related buckling phase, $L_{cr}$ is the buckled distance in the buckled zone, and I is the radius of gyration around the corresponding axis.

### 3.2. DASt-Richtlinie 015

As per DASt-Richtlinie 015 [29], the flexural resistance might be estimated using Equation (8), but with $\gamma_{M}$ instead of $\gamma_{M0}$, and the effective width of the compression flange determined using Equation (9).

(8) $$M_{Rd}=Min\;\{\begin{array}{c} \frac{f_{yf}*b_{cf,\;eff}*t_{cf}}{\gamma_{M}}*\;\left(h_{w}+\frac{t_{cf}+t_{tf}}{2}\right)\\ \frac{f_{yf}*b_{tf}*t_{tf}}{\gamma_{M}}*\;\left(h_{w}+\frac{t_{cf}+t_{tf}}{2}\right) \end{array}$$

(9) $$b_{cf,\;eff}=30.7*\;t_{cf}*\sqrt{\frac{240}{f_{yf}}}\;\leq \;b_{cf},$$

where *b_cf_* is the compression flange’s overall width. In order to prevent flange buckling, Equation (10) is also utilized to calculate the trapezoidal web’s maximum slenderness [29].

(10) $${\bar{\lambda }}_{pw}=0.80*\frac{h_{w}}{t_{w}}*\sqrt{\frac{f_{yw}}{E}*\frac{1}{K_{\tau }}\;}\leq \;{\bar{\lambda }}_{pw,\;max}=0.316*\sqrt{\frac{E}{f_{yw}}},$$

where *t_w_* represents the web thickness, *E* represents the modulus of elasticity, and *k_τ_* represents the coefficient of buckling due to shear which is equal (5.34).

The equivalent bending resistance of each tested beam may be estimated by entering the size and attributes of the tested specimens (S90B60h40, S80B80h40, and S80B80h50) into the aforementioned mathematical formula. Table 4 shows the computed moment of each specimen.

The study’s findings in Table 4 show that the EN 1993-1-5 [26] bending resistance equations may accurately predict specimen test data with a margin of safety ranging from 2% to 19%. On the other hand, the DASt-R015 [29] standard improperly predicted the test results and reported a greater bending resistance value in the 19–48% range.

## 4. Numerical Simulation

### 4.1. Modelling, Mesh Sensitivity, and Initial Imperfection

An FEM was created using ANSYS version 19.2 [30] to investigate the influence of the shear and bending spans on the capacity of the CWSBs loaded over the HFs. Two types of analyses were employed: an elastic buckling study was performed on a perfect web configuration to identify the initial mode shape of buckling, and a nonlinear static analysis was conducted to assess the performance and maximum moment of CWSBs. Newton–Raphson iterative algorithms contained in the application were used to continually update the element stiffness matrix. As a result, the stiffness matrix is updated at the end of each load step to represent the changes in stiffness until the model approaches the ultimate load. Corrugated steel webs, steel flanges, and welded stiffeners were represented using the four-node structural shell element SHELL181. Every node of the element has three degrees of freedom in translation and rotation in the horizontal, vertical, and out of plan dimensions. The elements’ properties include plasticity, large deflection, and high strain capacities. To improve the accuracy and convergence of the simulation findings, the test specimens’ FEMs were loaded under line load on the top of the beam, as in the experimental study.

Jáger et al. [24] advocated using six-to-ten elements per fold width when using a four-nodded shell (SHELL181) for satisfactory accuracy. Meanwhile, Kövesdi et al. [21] advised employing eight-to-ten components along the fold width for a precise assessment of the flange stresses. To ensure the numerical model’s accuracy, Alharthi et al. [15] performed a mesh sensitivity analysis to estimate the required number of elements per flange and fold widths—therefore eight elements per flange breadth—whereas for horizontal fold width variable numbers implemented based on fold length were employed in this work using a mesh size of 25 mm × 25 mm as shown in Figure 6b.

In this experiment, the mechanism of failure of the CWSB was impacted by local flange or shear buckling. As a result, the initial buckling mode was regarded as a defect in the model that was used to validate the FEMs and create numerical parametric studies. EN 1993-1-5 [26] outlines the initial imperfection value that is taken into account. An evaluation of *c_f_*/50 as an equal geometric imperfection is suggested in Annex C. With a Young’s modulus of 200 GPa, the material model follows Hook’s law and behaves linearly elastic up to the yield stress (*f_y_*). The yield plateau may be anticipated up to 1% strains with a minor increase in stresses. After reaching the yield strength, the material model exhibits an isotropic hardening behaviour with a hardening modulus until it reaches the ultimate strength (*f_u_*). Beyond this point, the material is regarded as entirely plastic, as seen in Figure 6a. Elgaaly et al. [1] used simple supports for beams loaded by the forces generated due to bending actions at the two ends of the as boundary conditions, and the proposed constraints were adequate for the experiments’ setting in this investigation. As a result, the anticipated boundary conditions are depicted in Figure 6b and are stated in Table 5. In [9,15], the current authors provided further details on the material and boundary conditions models.

**Figure 7:**
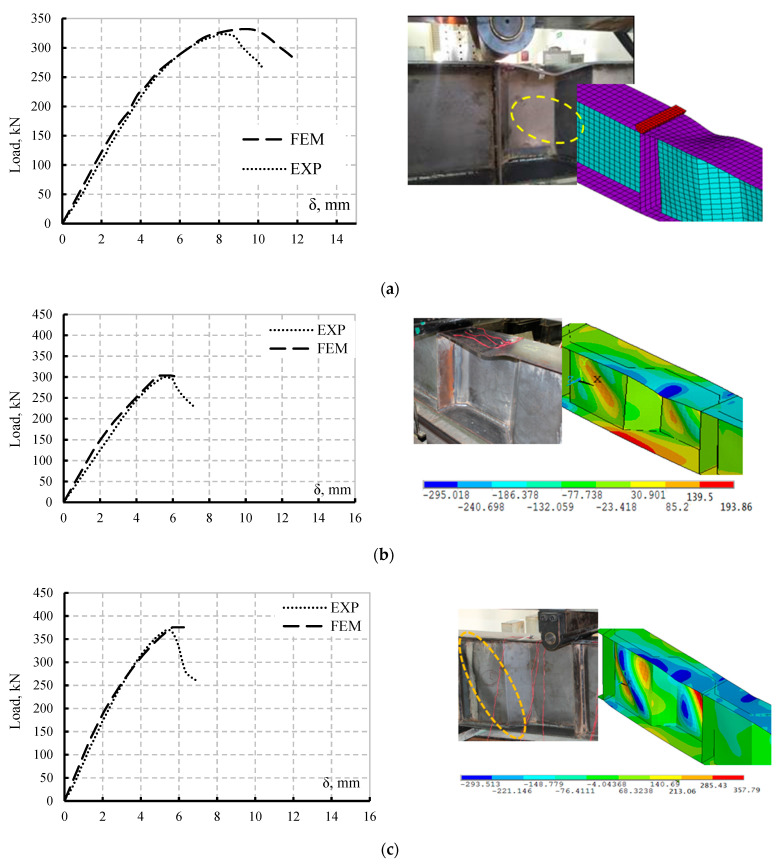
Experimental and FEM results comparison: load–vertical displacement curves and mode of failure; (**a**) Specimen S90B60h40; (**b**) Specimen S80B80h40; (**c**) S80B80h50.

To validate the FEMs, the ultimate loads, load–displacement responses, and the mode of failure of each specimen S90B60h40, S80B80h40, and S80B80h50 were compared with the results shown in (Figure 7). The experimental load–displacement responses and failure modes are quite consistent with the nonlinear FEM results.

### 4.2. Parametric Study

**Figure 8:**
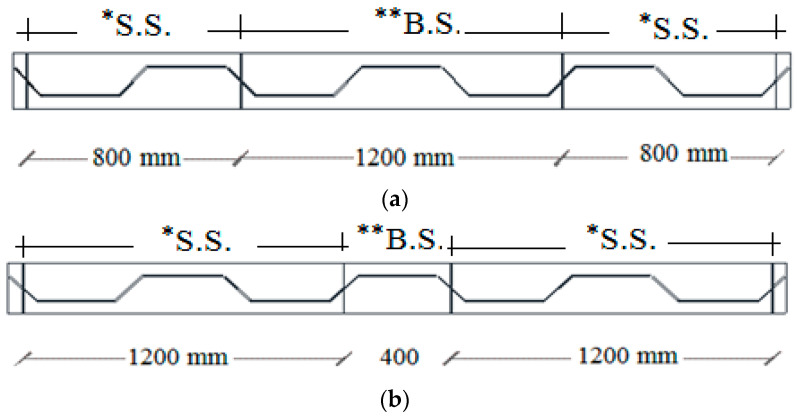
(**a**) SCWB—Shear span 0.8 m and Pure bending span 1.2 m, (**b**) SCWB—Shear span 1.2 m and Pure bending span 0.4 m. (* S. S. = Shear Span; ** B. S. = Pure bending span).

To examine the influence of pure bending and shear spans on the failure mechanism and ultimate load of SBCW, the following analysis was performed. The author proposed four new SBCWs models. The shear span in the first modelled SBCWs was 800 mm, while the pure bending span was 800 mm; then the bending span was increased by 50% for the second modelled SBCWs (B.S = 1200 mm, Figure 8a). Moreover, the third modelled SBCWs had a pure bending span of 400mm (half corrugation profile) and a shear span of 800 mm, then the shear spans were raised by 50% for the fourth modelled one (S.S = 1200 mm, Figure 8b). In the four simulated models, the same flanges, web properties and dimensions are employed. The aim of building these models is to study bending and shear spans’ influence on SBCW’s overall behaviour (i.e., failure mechanism, ultimate load, and load deflection curve).

**Figure 9:**
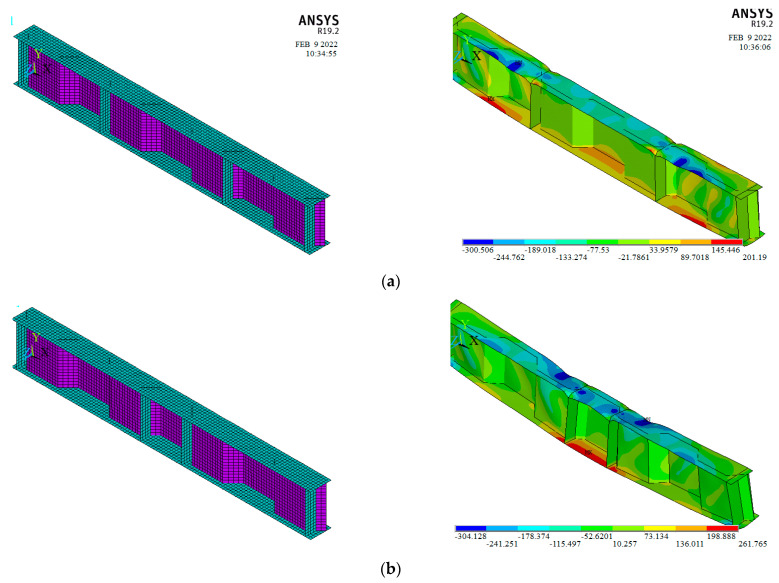
Finite Element Models and stress contour of proposed SBCWs (**a**) S80B120h40, (**b**) S120B40h40.

**Figure 10:**
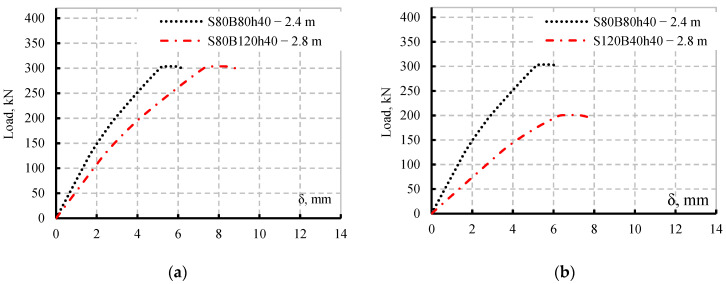
Pure bending and shear spans variable effects on Load vs. deflection curves; (**a**) bending span effect, (**b**) shear span effect.

Figure 9 illustrates the failure modes of the two modelled SBCWs (S80B120h40 and S120B40h40). From these results, it is concluded that increasing in shear span decreasing the ultimate capacity and increasing the deflection. Whereas, increasing the pure bending span increased the deflection only. Figure 10 shows a comparison between the load defection curve for simulated specimen S80B80h40 with both the simulated SBCWs (S80B120h40 and S120B40h40).

Figure 10a illustrates that increasing the pure bending span causes a greater deflection at a given degree of loading while maintaining a constant shear span results in a constant ultimate load. However, Figure 10b showed that increasing shear span results in a reduction in ultimate load and an increase in vertical deflection. The yield load for models S80B120h40 and S120B40h40 according to finite element models was between 80% and 85% of the ultimate load, and both models failed owing to local flange buckling. Furthermore, twelve finite models were developed and analysed to assess the previous findings. The models were built to simulate the behaviour of specimens S90B60h40 and S80B80h40 when various shear and bending spans were implemented. The newly constructed models were separated into two groups, the first group studying the shear span effect for specimens S90B60h40 and S80B80h40 while the second studying the pure bending span effect (Table 6).

**Figure 11:**
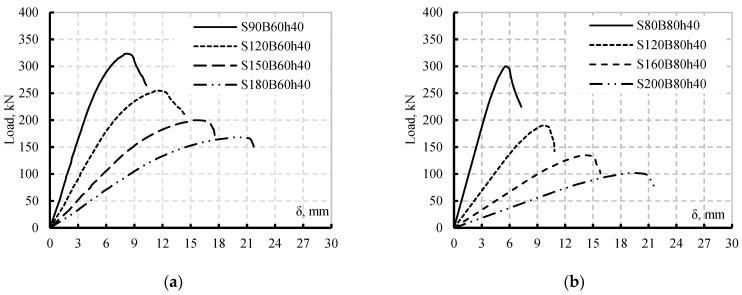

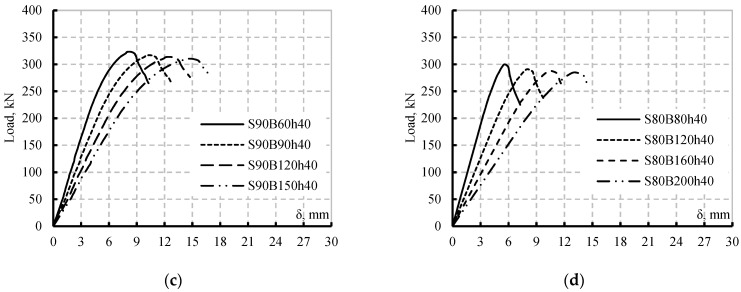
Comparison Load vs. deflections; (**a**) Group 1 Specimen S90B60h40, (**b**) Group 1 Specimen S80B80h40, (**c**) Group 2 Specimen S90B60h40, (**d**) Group 2 Specimen S80B80h40.

The results of the new simulated twelve beams compared to each controls ones are listed in Table 7. All the simulated SBCWs suffered from FB. Moreover, the loads capacities of the SBCWs decreased as S.S or B.S increased. The increase of the shear span had higher effects than increasing pure bending span on the load capacity reduction (Table 7). The two beams S120B60h40 and S90B120h40 having the same span lengths and different shear spans is an example. Moreover, increasing the shear span for beam S90B60h40 from 900 to 1800 mm decreased the load capacity by 48% while increasing the B.S from 600 to 1500 mm decreased the load capacity by only 4% (Table 7). The changes in B.S and S.S for beam S80B80h40 showed the same load trends as S90B60h40. Increasing the S.S of beam S90B60h40 from 800 to 2000 decreased the load capacity by 66 % while increasing B.S from 800 to 2000 decreased the load capacity by only 5%. In addition, beams S120B80h40 and S80B160h40 had the same span length, increasing the S.S with respect to B.S had a great effect on reducing the beam load capacity (Table 7). Figure 11 shows the load vs. deflection for the twelve simulated beams compared to their control ones. The figure assumed the higher effects of increasing the S.S on the beam’s stiffness than increasing the B.S for either beam S80B80h40 or S90B60h40. The previous findings assessed the effect of changing the S.S to B.S on accelerating the failure modes of the SBCWs, as the failure was FB.

## 5. Conclusions

Experimental, theoretical and analytical studies were conducted to examine the effect of shear and bending spans on the ultimate capacity and failure mode of SBCWs. Three beams with shear-span-to-effective-depth-ratios of 1.65 to 2.5 were evaluated under four-point bending to study the interaction between shear and bending spans and failure causes. The finite element approach was utilized to build finite element models for the tested SBCWs using ANSYS software. The maximum flexural that may be achieved for each specimen according to EN 1993-1-5 and DASt-Rishtlinie015 standards equations is calculated and compared with the values recorded experimentally. The findings showed that:

1. The ultimate load capacity and failure mechanism of SBCWs are not affected by pure bending span in both scenarios of loading (i.e., over inclined or horizontal folds); since the bending moment value does not change;
2. Shear span is the most important component in determining the ultimate load capacity of the SBCW. On the other hand, shear span can only influence the failure load value, whereas the failure mode is the same and controlled by the maximum bending moment that the compression flange can withstand;
3. The overall bending moment produced by the load multiplied by the moment arm, as well as the transversal bending moment formed by shear force, control the failure mode for SBCW governed by flange buckling modes;
4. The increase in S.S lengths had higher effects than increasing the B.S lengths on the beam capacity and stiffness; in contrast there were no effects on the failure modes.

## Figures and Tables

**Table 1:** Dimensions of fabricated specimens.

Specimen ID	Shear and Bending Span (mm)		CW (mm)			*h_w_* (mm)	*t_w_* (mm)	*b_f_* (mm)	*t_f_* (mm)
	* S. S.	** B. S. Loads	*b*	*d*	*h_r_*				
S90B60h40	900	600	200	100	100	400	3	200	8.0
S80B80h40	800	800	300			400			
S80B80h50	800	800	300			500			

* S. S. = Shear Span; ** B. S. = Pure bending span; *h* = beam height, *t_w_* = web thickness, *b_f_* = flange width, *t_f_* = flange thickness, *b* = horizontal fold, *d* and *h_r_* = horizontal projection and depth of inclined fold respectively.

**Table 2:** Modulus of elasticity (*E*), total strains, and ultimate (*f_u_*) and yield (*f_y_*) stresses.

Coupon Type	Average *f_y_* (MPa)	Average *f_u_* (MPa)	Average *E* (GPa)	Maximum Strain (−)
Web	352	418	198	0.075
Flange	270	360	192	0.102

**Table 3:** Experimental test results.

Beam ID	Loads and Deflection		Failure Mechanisms				
	*P_u_*	*δ_u_*	Web			Flange	
	kN	mm	Mode	Position	Angle	Mode	Position
S90B60h40	323	10.25	--	--	--	LB *	M + V
S80B80h40	305	5.58				LB	M + V
S80B80h50	365	4.52	LB	M + V	30°		

LB * = Local Buckling.

**Table 4:** Comparison between experimental and theoretical results.

Specimen	*M_exp_* (kN.m.)	EN1993-1-1		DASt-R015	
		M (kN.m.)	$\frac{M_{EN}}{M_{exp}}$	M (kN.m.)	$\frac{M_{DAT}}{M_{exp}}\;(b_{\mathrm{eff}})$ Equation (10)
S90B60h40	145.35	117.10	0.81	172.48	1.19
S80B80h40	122	114.05	0.93	172.48	1.41
S80B80h50	146	143.14	0.98	216.48	1.48

**Table 5:** Model boundary conditions.

	Degree of Freedom- Displacement			Degree of Freedom- Rotation		
	D_x_	D_y_	D_z_	R_x_	R_y_	R_z_
AB	C	R	R	R	R	--
CD	C	--	--	--	R	--

Note: R = Restrained; C = Constrained.

**Table 6:** Details of the simulated beams in the parametric study.

Beams I.D	Ls (mm)	h (mm)	b (mm)	Shear Span (S.S) (mm)	Bending Span (B.S) (mm)	Test Factors
S90B60h40	2400	400	200	900	600	Control beam (CB_90_)
S120B60h40	3000			1200	600	Effect of S.S
S150B60h40	3600	400	200	1500	600	
S180B60h40	4200			1800	600	
S90B90h40	3000			900	900	Effect of B.S
S90B120h40	3600	400	200	900	1200	
S900B150h40	4200			900	1500	
S80B80h40	2400	400	200	800	800	Control beam (CB_80_)
S120B80h40	3200			1200	800	
S160B80h40	4000	400	200	1600	800	Effect of S.S
S200B80h40	4800			2000	800	
S80B120h40	2800			800	1200	
S80B160h40	3200	400	200	800	1600	Effect of B.S
S80B200h40	3600			800	2000	

Ls = beam span, h = web height, and b = flange width.

**Table 7:** Results of the simulated beams in the parametric study.

Beams I.D	Ls (mm)	S.S (mm)	B.S (mm)	Yield load (kN)	Max. load (kN)	µ_u_ (%)	δ_u_ (mm)	Failure Mode (−)
S90B60h40	2400	900	600	284.24	323.0	−	8.42	FB
S120B60h40	3000	1200	600	204.79	254.4	78.8	11.87	FB
S150B60h40	3600	1500	600	167.07	199.6	61.8	16.12	
S180B60h40	4200	1800	600	141.96	168.0	52.0	20.37	
S90B90h40	2700	900	900	259.53	316.5	98.0	10.61	FB
S90B120h40	3000	900	1200	260.04	313.3	97.0	12.83	
S900B150h40	3300	900	1500	254.28	310.1	96.0	15.01	
S80B80h40	2400	800	800	243.00	300.0	−	5.57	FB
S120B80h40	3200	1200	800	161.50	190.0	63.3	9.58	FB
S160B80h40	4000	1600	800	109.22	133.2	44.4	14.98	
S200B80h40	4800	2000	800	84.66	102.0	34.0	19.17	
S80B120h40	2800	800	1200	244.44	291.0	97.0	8.01	FB
S80B160h40	3200	800	1600	236.16	288.0	96.0	10.44	
S80B200h40	3600	800	2000	230.85	285.0	95.0	13.05	

µ_u_ = the load capacity of the new simulated beams compared to their control ones, FB = flange buckling failure.

## References

[1] Elgaaly M., Seshadri A., Hamilton R.W. (1997). Bending strength of steel beams with corrugated webs. J. Struct. Eng..

[2] Aschinger R., Lindner J. (1997). Zu besonderheiten bei Trapezstegtragern. Stahlbau.

[3] Abbas H.H., Sause R., Driver R.G. (2007). Analysis of flange transverse bending of corrugated web I-girders under in-plane loads. J. Struct. Eng..

[4] Elgaaly M., Hamilton R.W., Seshadri A. (1996). Shear strength of beams with corrugated webs. J. Struct. Eng..

[5] Abbas H.H., Sause R., Driver R.G. (2006). Behavior of corrugated web I-girders under in- plane loads. J. Eng. Mech..

[6] Sause R., Abbas H.H., Wassef W.G., Driver R.G., Elgaaly M. (2003). Corrugated Web Girder Shape and Strength Criteria. ATLSS Reports.

[7] Abbas H.H., Sauce R., Driver R.G. (2007). Simplified analysis of flange transverse bending of corrugated web I-girders under in-plane moment and shear. Eng. Struct..

[8] Lindner J. (1992). Zur Bemessung von Trapezstegtragern. Stahlbau.

[9] Elamary A.S., Alharthi Y., Sharaky I.A. (2021). Behavior of steel beams with different web profiles along the beam length. J. Constr. Steel Res..

[10] Elamary A.S. (2016). Cardiff theory: Web panel aspect ratio limits and their relation with inclination angle of membrane tensile yield strength. Int. J. Steel Struct..

[11] Lindner J., Aschinger R. (1988). Grenz schubtragfähigkeit von I-Trägern mit trapezförmig profillierten Stegen. Stahlbau.

[12] Lee S.C., Lee D.S., Yoo C.H. (2008). Ultimate shear strength of long web panels. J. Constr. Steel Res..

[13] Luo R., Edlund B. (1996). Shear capacity of plate girders with trapezoidally corrugated webs. Thin-Walled Struct..

[14] Yi J., Gil H., Youm K., Lee H. (2008). Interactive shear buckling behaviour of trapezoidally corrugated steel webs. Eng. Struct..

[15] Alharthi Y., Sharaky I.A., Elamary A.S. (2021). Numerical analysis of hybrid steel beams with trapezoidal corrugated-web non-welded inclined folds. Adv. Civ. Eng..

[16] Moon J., Yi J., Choi B.H., Lee H.E. (2009). Shear strength and design of trapezoidally corrugated steel webs. J. Constr. Steel Res..

[17] Elamary A.S., Alharthi Y., Abdalla O., Alqurashi M., Sharaky I.A. (2021). Failure mechanism of hybrid steel beams with trapezoidal corrugated-web non-welded inclined folds. Materials.

[18] Eldib M.E.A. (2009). Shear buckling strength and design of curved corrugated steel webs for bridges. J. Constr. Steel Res..

[19] Kövesdi B., Jáger B., Dunai L. (2016). Bending and shear interaction behavior of girders with trapezoidally corrugated webs. J. Constr. Steel Res..

[20] Elamary A.S., Alharthi Y.M., Hassanein M.F., Sharaky I.A. (2022). Trapezoidally corrugated web steel beams loaded over horizontal and inclined folds. J. Constr. Steel Res..

[21] Kövesdi B., Jáger B., Dunai L. (2012). Stress distribution in the flanges of girders with corrugated webs. J. Constr. Steel Res..

[22] Kövesdi B. (2010). Patch Loading Resistance of Girders with Corrugated Webs. Ph.D. Thesis.

[23] Koichi W., Masahiro K. (2006). In-plane bending capacity of steel girders with corrugated web plates. J. Struct. Eng. JSCE.

[24] Jáger B., Dunai L., Kövesdi B. (2017). Flange buckling behavior of girders with corrugated web part II: Numerical study and design method development. Thin-Walled Struct..

[25] Dabon M., Elamary A.S. (2006). Flange compactness effects on the behavior of steel beams with corrugated webs. JES. J. Eng. Sci..

[26] (2005). Eurocode 3: Design of Steel Structures, Part 1–5: Plated Structural Elements.

[27] (2001). Recommendations for Welding of Metallic Materials, Part 2 Arc Welding of Ferritic Steels.

[28] (2004). Metallic Materials-Tensile Testing—Part 1: Method of Test at Ambient Temperature.

[29] für Stahlbau D.A. (1990). DASt-Richtlinie 015, Trager mit schlanken Stegen.

[30] (2019). ANSYS® v19.2.

